# The Effects of Stability and Presentation Order of Rewards on Justice Evaluations

**DOI:** 10.1371/journal.pone.0168956

**Published:** 2016-12-22

**Authors:** Hyomin Park, David Melamed

**Affiliations:** 1 Department of Sociology, Sungkyunkwan University, Seoul, Republic of Korea; 2 Department of Sociology, The Ohio State University, Columbus, OH, United States of America; Tianjin University of Technology, CHINA

## Abstract

Justice research has evolved by elucidating the factors that affect justice evaluations, as well as their consequences. Unfortunately, few researchers have paid attention to the pattern of rewards over time as a predictor of justice evaluations. There are two main objectives of this research. First, it aims to test the effect of reward stability on justice evaluations. Based on justice theory and prospect theory, we assume that an under-reward at one time cannot be fully offset by an equivalent over-reward at another time. Therefore, in unstable reward systems the asymmetry of the effect of unjust rewards with opposite directions will produce a lower level of justice evaluations over time. The second objective of this research is to show the moderating effect of the presentation order (primacy vs. recency) of unstable rewards on justice evaluations. The results from a controlled experiment with five conditions, which presents the instability of rewards in different orders, confirm both the negative effect of unstable rewards and the stronger effect of primacy on justice evaluations.

## Introduction

Justice theories postulate that individuals produce justice evaluations regarding their own or others’ rewards by comparing between actual and expected rewards [1, 2]. Because individuals in groups have similar just rewards in given situations, their expected rewards are often viewed as just rewards [3, 4]. If individuals’ actual rewards are incongruent with expected rewards, the rewards would be evaluated as unjust. Empirical research shows that as the incongruence between expected rewards and actual rewards becomes severe, justice evaluations proportionally decrease [1, 5]. Research on justice processes also reveals that justice evaluations are determined not only by objective reward levels (e.g. [6]), but also by factors such as individual characteristics (e.g. [7]) and situational factors (e.g. [8, 9, 10]).

Yet, most justice research has investigated justice processes at one specific time, implicitly assuming that justice evaluations are independent of each other. However, in many situations people make repeated justice evaluations of outcomes from the same source, making justice evaluations interdependent [11, 12]. Indeed, repeated justice evaluations are the rule rather than the exception, as wages from employment are among the most commonly researched “rewards.” Therefore, considering justice evaluations through time leads us to a more profound understanding of the justice process [13]. Recently, researchers have demonstrated the influence of reward stability on group commitment via the asymmetrical effects [14] of unjust rewards on justice evaluations [15].

Another issue with respect to repeated reward events is the effect of presentation order on justice evaluations. Research on judgment heuristics has consistently found that the position that information is presented (earlier or later) has an effect on judgment processes and leads to bias in decision making [16]. For example, Tversky and Kahneman [17] discovered the anchoring effect, which explains that judges can be disproportionately biased toward an initially presented value. Results showed that the group that was presented with numbers in ascending order estimated a lower outcome than the group that was presented with the same numbers in descending order. This suggests that the first number of the sequence operates as an anchor. Similarly, Markovsky [18] showed that similar biases can occur but in the opposite direction of the anchor, depending on the setting. Following these seminal studies, many researchers have tested anchoring effects in various situations other than laboratory settings. They have also shown the effect to be very robust in natural or uncontrolled situations (see [19] for further review).

Based on this, we examine the moderating effects of the “presentation order” of unstable rewards on justice evaluations. Several studies have shown a primacy effect in justice processes [20]. In this research, the focus is on the effect of the presentation order in the distributive justice process. In addition to this main objective, we also try to replicate prior findings of the effects of unstable rewards. Though the asymmetry between under-reward and over-reward has been predicted by justice theory [21], empirical tests on the effect through time have rarely been conducted until recently. These results have practical implications for reward distributions in groups, organizations, and more generally for employers.

## Reward Stability and Justice Evaluations

Social scientists have repeatedly found an asymmetry in the experience of negative and positive events; negative events have stronger effect on people’s perception than positive events [14, 22]. Similarly, prospect theory shows that losses have a greater effect on experiences than gains [23], especially when they use the same scale to assess those losses and gains [24]. Justice theories also posit that though both under-rewards and over-rewards produce negative feelings among individuals, reactions to under-rewards will be more pronounced than reactions to over-rewards [5, 21, 25–27]. More recent empirical research supports the asymmetric effect of voice on perceptions of procedural justice. Based on prospect theory and theories of procedural justice, Paddock and colleagues [28] show that members’ voice in decision making has a positive effect on justice perceptions in nine different cultures. Importantly, they show that loss of voice has a greater negative effect than an equivalent gain.

The results reported above lead us to predict that when a series of repeated rewards occurs through time, under-reward at one particular time cannot be fully compensated for by the same amount of over-reward at another time. Likewise, over-reward at one particular time can be canceled out by a smaller under-reward at another time. Through this process, the instability of a reward system has an overall negative effect on justice evaluations. In this research, we consider fluctuations of rewards through time as an important factor in shaping justice evaluations. Justice researchers have paid little attention to the history of repeated rewards in examining the principles of justice evaluations. Instead, most studies on distributive justice have treated an individual’s investments into a group, and rewards from the group, as a single event, and focused on the results of the allocation of rewards. Therefore, they have not accounted for reward stability. Given that most reward events are repeated through time, investigating rewards through time allows us to understand the justice process more profoundly [13]. Park and Melamed [15] recently tested the net effect of stable rewards in the area of distributive justice. Their results supported the theoretical prediction that unstable rewards decrease justice evaluations among group members, which, in turn, decrease group cooperation and commitment. Their research, to our knowledge, is the only empirical evidence of the effect of reward stability on justice evaluations. Furthermore, the experiment did not include baseline justice evaluations in a stable rewards condition. Due to this limitation, the net effect of an asymmetry between under-reward and over-rewards compared to stable rewards is unknown.

## Presentation order and justice evaluations

Another factor we focus on in this study is the effect of the presentation order of the instability of rewards. The order that people experience events shapes their judgments [29]. Research on primacy effects maintains that information presented first has a stronger effect on judgments and is more likely to change an individual’s judgment than information that is presented last [30, 31]. On the other hand, other researchers have found a recency effect, which suggests that information presented last has a stronger effect than the information that is presented first [32, 33]. Though the evidence is contradictory, both the primacy and the recency effects demonstrate the power of serial positioning effects.

Prospect theory and justice theory also suggest that the starting point or anchor plays an important role in producing justice evaluations. The concept of an “endowment effect” explains that individuals place a higher value on the goods that they already possess, while devaluing the goods of others in exchange relations [34]. Tversky and Kahneman [17, 35] also found an anchoring effect on judgments. Specifically, they found that variation of the reference point of judgments can change the evaluations of gains and losses. Later, Markovsky [18] argued that justice evaluations are shaped by social contexts or framing information. Using vignette experiments, he showed that justice evaluations could be biased either toward (assimilation) or away from (contrast) the anchor when the anchor is salient in the situation. Furthermore, the study shows that the information presented first can serve as an “anchor” for the information that follows.

In the procedural justice literature, researchers have repeatedly found primacy effects on the justice process. Based on fairness heuristic theory [36], research has found that information about the fairness of the situation has a stronger effect on people’s justice evaluations when it appears at an earlier stage of an interaction than at a later stage [20, 37]. These results are also stable across different cultural environments.

Based on the extant research reviewed above, the most important of which is the research on anchoring, the current study assumes that the order of unfair rewards also has a net effect on justice evaluations. Based on this work, we hypothesize that justice evaluations are affected more strongly by reward events that come first (primacy effect). Therefore, the instability of rewards at an earlier point in time will have a stronger effect on justice evaluations than unstable rewards of the same degree that come later. By contrast, if recently experienced reward events affect justice evaluations more strongly (recency effect), the instability of rewards that come later will have a stronger effect on justice evaluations. We therefore expect the following:

*Hypothesis 1*: The negative effect of unstable rewards on justice evaluation is stronger when they appear earlier.
*Hypothesis 1a*: The effect of the instability of rewards on justice evaluation is stronger when unstable rewards appear earlier than stable rewards.
*Hypothesis 1b*: The effect of the instability of rewards on justice evaluation is stronger when under-rewards appear earlier than over-rewards.

A second purpose of our experiment is to replicate the results from previous research on the net effect of unstable rewards on justice evaluations [15]. Since this is a nascent area of study, it is important to replicate the finding in multiple experiments to confirm the effectiveness and robustness of the stability of rewards.

*Hypothesis 2*: The stability of the reward structure is positively related to justice evaluations.

Our argument can be summarized as follows: (1) unstable reward structures are negatively related to justice evaluations (i.e., unstable structures yield less fair evaluations), (2) the effect of instability of reward structure on justice evaluations is moderated by the presentation order such that unstable rewards experienced early on have a stronger effect than unstable rewards experienced later.

## Methods

### Ethic Statement

This study has been approved by the Health Sciences South Carolina (HSSC) electronic Institutional Review Board (eIRB; Approval No. Pro00020785) and has been conducted in full accordance with the World Medical Association’s Declaration of Helsinki. All participants signed an informed consent form before participating in the experiment. The consent form was presented on a PC screen and saved separately from the data files. The consent procedures were also approved by the HSSC and the data from the experiment were collected and analyzed anonymously.

**Experimental Design** (see S1 Text for the instructions and details of the experiment)

150 undergraduates from a large public university in the U.S. participated in the experiment. For testing explicit theory, homogeneous populations (e.g., undergraduate students) are optimal as they minimize the likelihood of falsely rejecting a true null hypothesis. Subsequently, the theory can be generalized (but not the results) to other situations [38, 39]. During the experiment subjects were placed in isolated rooms with personal computers on which they completed the experiment. The experimental protocol was completely computer mediated to minimize any interaction between the participants and the experimenter. The subjects were informed that the study addresses reward satisfaction in a group task and then were asked to complete a consent form. They were also asked to take part in a controlled group task that guaranteed more profits through collaboration than through individual effort.

The cover story stated that the participants were in a four-person task group in a computer programming company. The group consisted of one manager (M) and three programmers (P1, P2, and P3). The subjects were told that each subject was randomly assigned to either the manager’s role or to one of the three programmers’ roles. However, all the subjects were assigned to be a programmer (P2), and the manager and the other programmers were simulated by the computer program. This enabled us to control the behaviors of the other group members, ensuring that every participant experienced the exact same thing.

Programmers were given a pool of resources at the beginning of each round (ostensibly converted into cash at the conclusion of the experiment), and they could decide whether to invest or keep the resources. Programmers could contribute anywhere between 0 and 500 resources per round to the collective effort. According to the company’s guidelines, the manager was supposed to return 1.3 times the investment made by each programmer and keep 0.2 times the investment for profit. If a programmer contributed 200 resources, for example, she should expect 260 back from the manager. However, the programmers were told that, despite the guidelines, their rewards would be decided by their manager at the end of every contribution opportunity. That is, although the manager was supposed to distribute rewards based on the company’s guidelines, the final decisions were actually up to the manager. The manager’s final decisions were simulated by predetermined parameters using a computer program (see below for the details). The subjects have no voice in the reward allocation process, but they could express their reactions to their reward levels by answering the questions presented after each investment opportunity. The participants could also decide the amount of resources they invested in subsequent investment opportunities.

At the end of the instructions, several quizzes were administered to make sure that the subjects understood the structure of the experiment. Then the subjects participated in the investment opportunities (exchange sessions). The subjects engaged in 18 rounds of investment-reward trials. To avoid end game effects, they did not know how many trials there would be. Each experiment took about 30–60 minutes for subjects to complete.

The experiment consisted of five conditions. The experimental conditions manipulated the instability of rewards in two ways (2 × 2): the order of unstable rewards (primacy vs. recency) and the order of incongruence (under-reward first vs. over-reward first). To establish a baseline of justice evaluations, we added a control condition, which did not include any incongruence of rewards throughout the experiment.

### Stability Manipulation

**Fig 1:**
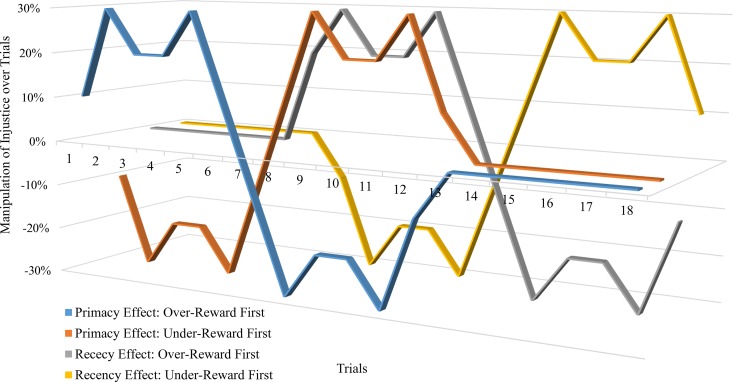
Manipulation Schedule of Conditions.

Prior to starting the group task, subjects were informed in detail about the processes that determined reward levels, and they were told to expect 1.3 times higher rewards than their investments in each round of the productive exchange task. In the stable reward condition, rewards from the company, as allocated by the ‘‘Manager,” meet the reward expectations; therefore the participants should evaluate the rewards as just. In the experimental conditions, however, reward levels deviated from the expected reward level between 10 percent and 30 percent, either positively or negatively, according to predetermined parameters. It is important to note that in the unstable conditions, at the end of the group task, the overall reward level was the same as the expected level (Fig 1).

In the primacy conditions (conditions 1 and 2), twelve unstable rewards were presented first and were followed by six stable rewards. In the recency conditions (conditions 3 and 4), stable rewards appeared in the first six rounds and unstable rewards were presented in the next twelve rounds. In the unstable reward rounds, the rewards varied from the expected reward level by 10–30% (on average 20%).

Each of the primacy conditions and recency conditions consisted of two sub-conditions: an under-reward-first condition and an over-reward-first condition. In the under-reward-first condition, rewards from the first six rounds among the twelve unstable reward rounds were *lower* than the expected level, and the rewards fluctuated *above* the expected level in the following six rounds. In the over-reward-first condition, the rewards fluctuated *above* the expected level in the first six rounds and were followed by six rounds in which the rewards fluctuated *below* the expected level. In the control condition, the rewards were stable throughout the experiment.

If the primacy effect is prominent, as hypothesized, the justice evaluations in conditions 1 and 2 will be lower than the justice evaluations in conditions 3 and 4 (*Hypothesis 1a*). In addition, the justice evaluations in the over-reward-first conditions (conditions 2 and 4) will be higher than the justice evaluations in the under-reward-first conditions (conditions 1 and 3) (*Hypothesis 1b*). Therefore, it was predicted that justice evaluations are highest in condition 4, followed by conditions 3, 2, and 1. When it comes to the effect of the stability of rewards (*Hypothesis 2*), justice evaluations in the control condition (condition 5) should be higher than the justice evaluations in the other four experimental conditions (conditions 1–4).

We measured justice evaluations with a 10-point scale as a main dependent variable after each investment had been returned (See S1 Text for the wording of the scale). In addition, participant’s gender, age, race, and year in school were measured. Since trials are nested in each subject, random intercept models were used for the analyses. Specifically, using an individual growth model [40], the analyses decompose fixed and random effects using a maximum-likelihood estimator. Preliminary analyses for model specification showed that the background variables did not have significant effects (see S1 Table for the model specifications).

## Results

The analyses come from data from 150 participants. A total of 164 participants were recruited in the experiment and 14 were excluded from the analyses for reporting being suspicious or not understanding the manipulations. The participants were randomly distributed across the five conditions. Each condition has 30 participants, and each participant completed 18 rounds in the experiment, making a total sample of 2,700 participant-rounds. Table 1 shows the descriptive statistics for the control variables: subjects’ gender, age, race, and year in college.

**Table 1:** Descriptive Statistics of the Participants’ Demographics.

	Mean	Std. dev.	Min	Max
Female	.500	–		
Age	20.407	2.167	17	34
White	.640	–		
College Year	2.533	1.094	1	5

**Fig 2:**
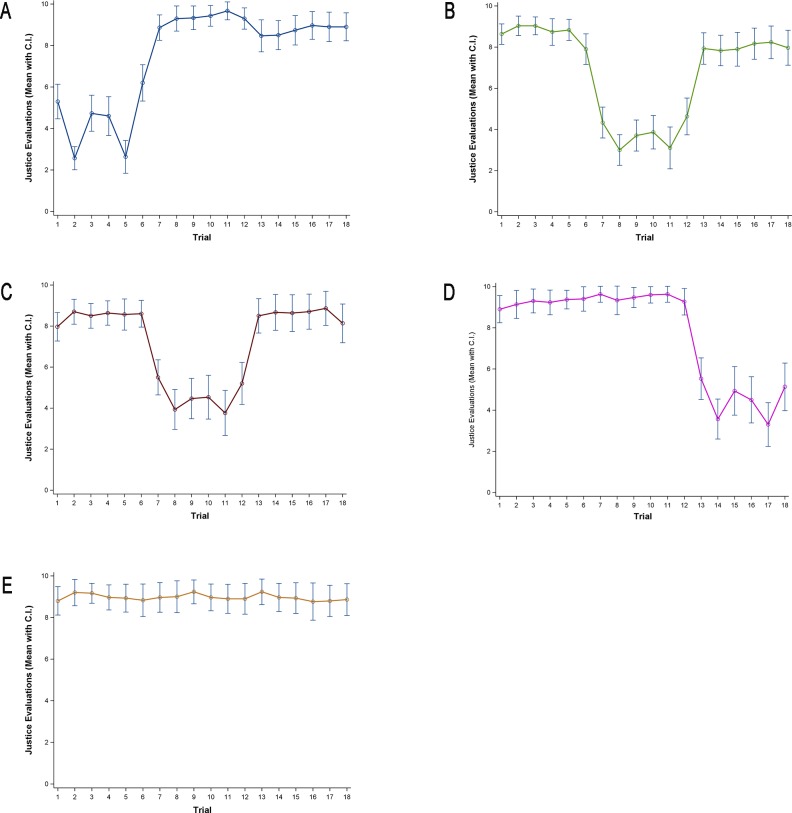
Justice Evaluations by Trials. Each panel presents justice evaluation levels in (A) Under-Reward/Primacy Condition, (B) Over-Reward/Primacy, (C) Under-Reward/Recency Condition, (D) Over-Reward/Recency Condition, and (E) Control Condition. In all graphs, the horizontal axis indicates “Trial” and the vertical axis indicates “Justice Evaluations (mean with 95% confidential interval)”.

Fig 2 plots a trend of the means with confidence intervals of justice evaluations over trials in each condition. The graph shows that the justice evaluations reflect the reward manipulations over trials. In particular, the justice evaluations in under-reward trials exactly follow the manipulation schedule. However, the differences in justice evaluations between justly-rewarded and over-rewarded trials are not as large as the differences in justice evaluations between justly-reward and under-rewarded trials (compare Fig 1 and Fig 2).

**Fig 3:**
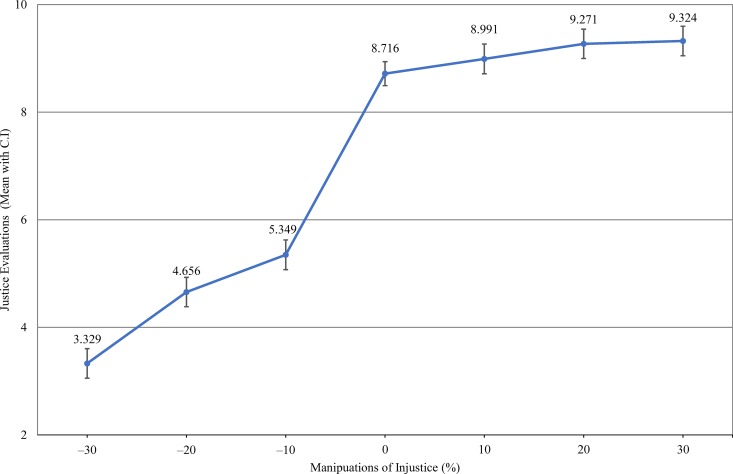
Justice Evaluations across the Level of Manipulations.

Fig 3 show the asymmetric tendency more clearly. The justice evaluations across different reward levels show a negative effect of under-rewards (p < .001 for all under rewards), and a positive effect of over-rewards (when over-rewarded by 10% the difference is not significant (p = .106), when over-rewarded by 20% or 30%, p < .01) on justice evaluations. This result replicates prior research on asymmetries in justice evaluations, with under-reward being experienced more profoundly than over-reward [15, 28].

To test the effect of reward stability, we estimated a linear mixed model with trials nested in participants (i.e., a random intercept model). The model specified the effect of the presentation order of unstable rewards (primacy vs. recency), the presentation order of unjust rewards (under-reward-first vs. over-reward-first), and the interaction of the two manipulations. Omnibus tests from the mixed model revealed a significant effect of the presentation order of unstable rewards (*F*_(1, 145)_ = 22.96, *p* < .001), the presentation order of unjust rewards (*F*_(1, 145)_ = 8.27, *p* = .005), and the interaction between these two manipulations (*F*_(1, 116)_ = 5.94, *p* = .016) on justice evaluations.

Comparing between conditions shows that the estimated justice evaluations are higher in the recency conditions (conditions 3 and 4) (*Mean* = 7.555) than the primacy conditions (conditions 1 and 2) (*Mean* = 6.671) (*t*_(116)_ = –4.79, *p* < .001). That is, unstable rewards have a stronger negative effect on justice evaluations when they appear earlier in a series of rewards than when they appear later. In the case of justice evaluations through time, primacy is stronger than recency. This result supports hypothesis 1a. Furthermore, the results show that the over-reward-first conditions (conditions 1 and 3) yield higher justice evaluations (*Mean* = 7.379) than the under-reward-first conditions (condition 2 and 4) (*Mean* = 6.848) (*t*_(116)_ = –2.88, *p* = .005). This result suggests that unstable rewards have a stronger negative effect when they are presented in an earlier stage of a reward sequence, and this supports hypothesis 1b.

Table 2 presents the main and interaction effects of the manipulations on justice evaluations. The coefficients confirm that the recency manipulation and over-reward-first manipulation have positive effects on justice evaluations, as stated above. When it comes to the interaction effect, the result shows a positive interaction effect between the manipulations (*β* = .898, *p* < .01); that is, the effect of the over-reward-first manipulation is stronger in the recency effect conditions than in the primacy effect conditions.

**Table 2:** Estimated Fixed Effects of the Manipulation and Interaction.

	*β*	Std. Err.	*t*- value
Interception	6.631	.184	43.63
Recency effect^1^	1.333	.261	5.11
Over-Rewards-First effect^2^	.980	.261	3.76
Recency × Over-Rewards First	.898	.369	2.44

^1^ The primacy conditions are the reference category.

^2^ The under-reward first conditions are the reference category.

**Fig 4:**
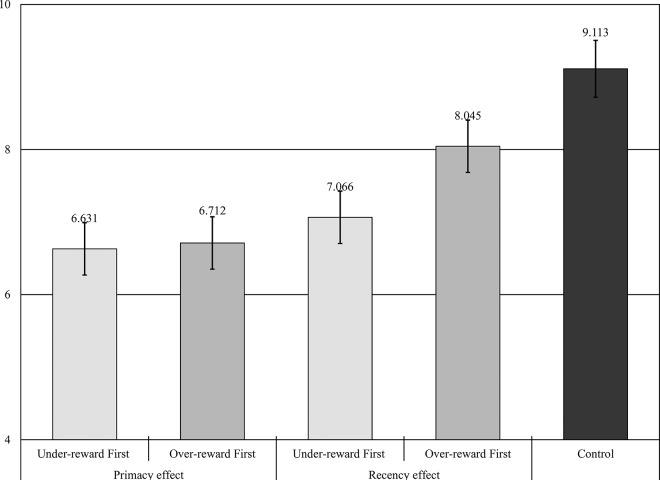
Estimated Justice Evaluations across the Conditions.

Fig 4 presents the justice evaluations for each experimental condition and the control condition. Consistent with previous results, among the experimental conditions justice evaluations were highest in condition 4 (*Mean* = 8.045) and second highest in condition 3 (*Mean* = 7.066). Between the primacy conditions, condition 2 (*Mean* = 6.712) yields higher justice evaluations than condition 1 (*Mean* = 6.631).

Lastly, to replicate the results of prior work we tested whether the effect of reward stability on justice evaluations is positive. To do this, the justice evaluations between the experimental conditions and the control conditions were compared. An omnibus test from the mixed model revealed a significant effect of condition (*F*_(1, 148)_ = 77.58, *p* < .001) and reward level (*F*_(1, 148)_ = 850.15, *p* < .001) on justice evaluations. A comparison between the control condition and the experimental conditions revealed that the control condition (*Mean* = 9.113) showed higher justice evaluations than the experimental conditions (*Mean* = 7.145) (*t*_(116)_ = 8.81, *p* < .001), revealing that reward stability has a positive effect on justice evaluations (hypothesis 2). What is more, the control condition also yields higher justice evaluations than the fairest experimental condition (*t*_(58)_ = 10.24, *p* < .001).

## Discussion

In everyday life, exchange relations between individuals are not one-time transactions, but instead, individuals more often belong to groups and repeat exchange relationships with others in the same groups. From relationships with intimate partners or close friends to economic transactions, individuals frequently sustain repetitive relationships. In those situations, individuals’ justice evaluations about their groups are not independent but are highly contingent upon each other. Therefore, investigating justice processes in a repeated reward framework more accurately reflects the world.

When it comes to justice processes, researchers have found that justice evaluations are open to biases from a variety of factors (Berger, Ridgeway, and Zelitch 2002, Hegtvedt and Johnson 2009, Morris and Leung 2000). In this research, the presentation order of rewards was the main factor being addressed. The results from the experiment show that unstable rewards are associated with lower justice evaluations, and that the presentation order of unstable rewards also matters; the primacy effect is more prominent than the recency effect (e.g. [28]). The results also demonstrate the asymmetric effects between under-rewards and over-rewards. Prior research in justice theory found a logarithmic function between reward levels and justice evaluations [1, 5]. In those studies, over-rewards decrease justice evaluations, though the steepness decreases as the extent of over-rewards becomes larger. This research assumes that the negative effect of reward instability on justice evaluations comes from the asymmetry of unjust rewards. With respect to this assumption, our results showed that the effect of under-rewards is stronger than the effect of over-rewards of the same size. This result replicates a recent test of the asymmetry of justice evaluations in repeated reward events [15].

We also tested the effect of the presentation order of unstable rewards on justice evaluations. This study supported the primacy effect of the instability of rewards. The results showed that the negative effect of unstable rewards is more pronounced when the unstable rewards are presented earlier than stable rewards. Furthermore, unstable rewards have a stronger effect when under-rewards are presented prior to over-rewards. These results are quite consistent with previous research on the effect of presentation order in justice evaluations [20]. Taken together, this research suggests that stable reward structures yield the most fairness and satisfaction, and that unstable reward structures with initial under-rewards yield the least.

These results have practical implications for reward distributions at the group and organizational levels. For instance, we can predict that failure to maintain a stable reward system will decrease justice evaluations among employees. This has practical implications for cooperation and organizational commitment [15]. Moreover, this research shows that the negative effect will be stronger if the company gives a bad first impression regarding its rewards system. As we have seen, it is harder to recover from low justice evaluations caused by unjust / unstable rewards when the injustice occurs earlier rather than later. Our results also suggest that customer service agencies should focus more on customers’ early-stage experiences with the company or their products in order to keep the customers’ loyalty high because bad experiences earlier on can only be cancelled by an increased amount of positive experiences later in time. In sum, in a repeated exchange relationship, unstable rewards are bad; starting with unstable rewards is worse, and starting with unstable under-rewards is worst of all.

Lastly, while we explicitly examined repeated reward encounters we did not investigate the net effect of time on the dependent variable. As previous research has shown, people’s behaviors in exchange relations changes over time, especially when they interact with the same partners repeatedly [41, 42]. Thus, future research on the focal phenomenon should include a time series analysis method to see how individual’s behavior changes over time as they get more information on their exchange partners. This will provide us with a more profound understanding of justice evaluations in unstable reward situations [43].

## Supporting Information

S1 Table Summary of Multi-level Models predicting Justice Evaluations. The tables present a series of model specifications which are used for analysis. The preferred model in each specification is highlighted in gray. Summary of Multi-level Models predicting Justice Evaluations (Table A). Specifying Covariance Structure of the Model predicting Justice Evaluations (Table B).
(DOCX)

S1 Text The Details of the Experiment. (DOCX)
