# Supplementary material for: The Effects of Stability and Presentation Order of Rewards on Justice Evaluations
Source: PLoS One. 2016 Dec 22;11(12):e0168956. doi: 10.1371/journal.pone.0168956 (PMC5179236; doi:10.1371/journal.pone.0168956)
Supplement: S1 Table — The tables present a series of model specifications which are used for analysis. The preferred model in each specification is highlighted in gray. Summary of Multi-level Models predicting Justice Evaluations (Table A). Specifying Covariance Structure of the Model predicting Justice Evaluations (Table B). (DOCX) [file pone.0168956.s001.docx]

**Supporting Information: Summary of Multi-level Models predicting Justice Evaluations**

Table A. Summary of Multi-level Models predicting Justice Evaluations

| Justice Evaluations | -2LL | # of Parameters | Chi^2^ | d.f. | *p*-value |
| --- | --- | --- | --- | --- | --- |
| Null Model | 10272.8 | 1 |  |  |  |
| Manipulation | 10035.7 | 2 | 237.1 | 1 | < .001 |
| Condition | 10213.6 | 6 |  |  |  |
| M^1^ + C^2^ | 9974.6 | 7 | 239.0 | 1 | < .001 |
| M + C + M×C | 9984.8 | 12 |  |  |  |
| Full Model | 9994.2 | 16 |  |  |  |

^1^ Manipulation of Rewards

^2^ Condition

Table B. Specifying Covariance Structure of the Model predicting Justice Evaluations

| Justice Evaluations | -2LL | # of parameters | Chi^2^ | d.f. | *p*-value |
| --- | --- | --- | --- | --- | --- |
| UN | 9974.6 | 171 |  |  |  |
| Ante(1) | 10557.0 | 35 | 582.3 | 136 | < .001 |
| AR(1) | 10759.2 | 2 | 784.5 | 169 | < .001 |
| ARH(1) | 10697.4 | 19 | 722.8 | 152 | < .001 |
| ARMA(1,1) | 10553.0 | 3 | 578.3 | 168 | < .001 |
| CS | 11436.2 | 2 | 1461.5 | 169 | < .001 |
| CSH | 11251.1 | 19 | 1276.5 | 152 | < .001 |
| TOEP | 10523.7 | 18 | 549.1 | 153 | < .001 |
| TOEPH | 10472.9 | 35 | 498.3 | 136 | < .001 |
| VC | 11971.1 | 1 | 1996.5 | 170 | < .001 |
