# Supplementary material for: The Effects of Stability and Presentation Order of Rewards on Justice Evaluations
Source: PLoS One. 2016 Dec 22;11(12):e0168956. doi: 10.1371/journal.pone.0168956 (PMC5179236; doi:10.1371/journal.pone.0168956)
Supplement: S1 Text — (DOCX) [file pone.0168956.s002.docx]

**Supporting Information: The details of the Experiment**

**1. Instructions for the Experiment**

We are members of a research team of social scientists who are interested in studying group cooperation. In today’s study, you will belong to a task group. In that group, you will be randomly assigned to the role of decider (manager) or contributor (programmer)

Let’s assume the following situation: There are freelance computer programmers who are now working alone. A computer program company finds out that there are demands for a bunch of new computer programs in the market and the programmers are able to develop the programs. The company suggest that the programmers develop the new programs together. It will guarantee better profits to the programmers than if developed and sell the program by themselves, because the company will advertise and ensure mass distribution. Thus, the programmers decide to work together in the company.

To develop the programs, the programmers will decide how much time and skill they will invest in the project. This amount will be represented by *“resource unit (RU)”* in this experiment. After they develop each program, the company sells it on the market on behalf of the programmers. Then the manager of the company will distributed the revenues on the programmers.

In this study, one group is composed of 4 participants who are randomly assigned to two different roles: a manger and a programmer. Only one participants will be assigned to the manager’s role. If you assigned to be a manager, you will not be involved in developing computer programs. But after each program is developed through the programmers’ investments, the manager will divided the revenue among the programmers according to the amount of work they invested in the program. As a manager, you will be informed of all programmers’ investments levels in the task. Thus, you can use this information in deciding reward allocations.

The rest of the participants will be assigned to a programmer’s role. If you are assigned to be a programmer, you are supposed to develop a new program in each session with your RUs(resource units) which represents your time and skills that are ready to invest. You will start every session with some initial RUs. After investment, you will get paid from the participant who is assigned to the manager’s role. You are only able to know about your own investment and reward information. The other programmer’s level of investments a rewards will be unknown.

Now, let’s begin with the study with assigning your role. Please wait while the experimenter is randomly assigning the roles of each participants…. You are assigned to a programmer’s role. You are programmer 2 (P2).

Let’s talk more about the programmer’s role. Each programmer has 500RUs in each session that can be invested to develop a new program. The products developed by you and other programmers will be sold in market by the manager’s effort, and will bring 1.5(150%) times higher revenue to the company. The participant who is assigned to be a manager is supposed to decide your payment. The company’s payment guideline recommends that the invested group RUs from the programmers will be multiplied by 1.3(130%) and returned back to the programmers. However, it is the manager who finally decides programmers’ payments, and your payment can vary according to the manager’s decision. The rest of the profit: *total revenue – programmers’ payment*, will be the payment for the manager.

While the manager has final say over payment amounts from investment, programmers can decide how much to invest from their RUs. You can keep the RUs not invested in the group task. The programmers will develop several different programs and will be asked to decide their investment in each time.

Importantly, your total RUs will be converted in to real money with at the end of the experiment and paid to you. That is, you will paid for the RUs that you do not invest and for your payments from investments throughout the experiment. The RUs you earn from this experiments will be rounded up to the nearest thousandth and converted to $1 per 1000 RUs. For example, if you earn 6200RUs, you will get $7.

**2. Quizzes (Bolds are the right answers)**

How many people are in your group?

1 person 3 people **4 people** 5 people

How many programmers are in your group?

1 programmer 3 programmers

**4 programmers**  5 programmers

You are assigned to be _______.

A manager **A programmer**

An experimenter Neither of them

According to the company’s guidelines, each programmer’s invested RUs will be multiplied by about ______ times and returned to the programmer.

1.0 times **1.3 times**

1.5 times 2.0 times

If you invest in 400RUs to develop the program, how much RUs would you expect to earn from the company?

360 RUs 400 RUs

**520 RUs** 600 RUs

**3. Questionnaire (Each set of questionnaire was administered after each trial)**

My Payment form the company were

**Very Unjust 1 – 2 – 3 – 4 – 5 – 6 – 7 – 8 – 9 – 10 Very Just**
